# Contrasting melt regime in the Ice Grounding Zone of Thwaites Glacier, West Antarctica

**DOI:** 10.1073/pnas.2512626122

**Published:** 2025-11-17

**Authors:** Ratnakar Gadi, Eric Rignot, Dimitris Menemenlis, Bernd Scheuchl

**Affiliations:** ^a^Department of Earth System Science, University of California, Irvine, CA 92627; ^b^Department of Civil and Environmental Engineering, University of California, Irvine, CA 92627; ^c^Radar Science and Engineering Section, Jet Propulsion Laboratory, California Institute of Technology, Pasadena, CA 91011

**Keywords:** Antarctica, southern ocean, sea level rise, grounding line, glaciology

## Abstract

The contribution of Thwaites Glacier, Antarctica, to sea level rise is influenced by how quickly warm salty seawater of Circumpolar Deep Water origin melts basal ice near its grounding line. Satellite observations reveal tidally forced kilometer-scale seawater intrusions beneath grounded ice that form an “Ice Grounding Zone” (IGZ) where ice melts vigorously. Although melt rates have been measured at selected sites using Automated phase-sensitive Radar Echo Sounders (ApRES) and Automated Underwater Vehicle (AUV) instruments, their spatial distribution and total magnitude within the IGZ remain uncertain. Here, we present 2D high-resolution simulations of the melt regime of Thwaites Glacier using the Massachusetts Institute of Technology global circulation model ocean model. The model predicts high melt at the entrance of the IGZ, with a quadratic decay inside the IGZ, a linear increase with ocean thermal forcing and a sublinear increase with cavity length. On the slow-moving Thwaites Eastern Ice Shelf, the modeled melt rate is low (10 m/y) due to a flat, shallow ice base, in agreement with in situ observations from AUV and ApRES instruments. On the Thwaites Glacier Tongue (TGT), the modeled melt rate is high (50 m/y) due to a steeper and deeper ice base. The modeled melt rates are consistent with the estimates derived from the satellite. Hence, multiple lines of evidence indicate high melt in the IGZ of TGT, which controls 80% of the glacier mass balance. An updated representation of grounding zones in ice sheet models including seawater intrusions will increase their sensitivity to ocean warming and will revise sea-level projections upward.

## Significance

The transition boundary between continental and floating ice, or grounding line, is traditionally represented as a fixed boundary experiencing zero ice melt. Satellite observations have challenged this view and revealed a kilometer-scale “Ice Grounding Zone” (IGZ) where ice melt is vigorous. Here, we use an ocean model to reconstruct the melt regime in the IGZ cavity versus the temperature of ocean waters and cavity geometry of Thwaites Glacier, Antarctica. The modeled melt rates compare well with in situ observations and satellite remote sensing estimates of rapid ice melt at the entrance of the IGZ. The inclusion of IGZ in ice sheet numerical models will increase their sensitivity to ocean temperature and increase the projections of sea level rise from Antarctica.

**Fig. 1.**
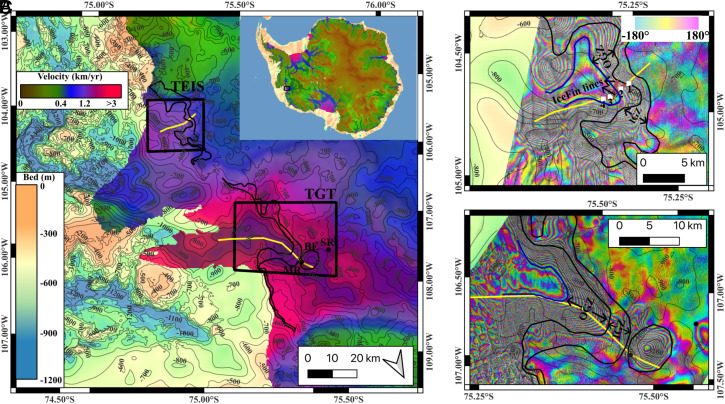
Ice Grounding Zone (IGZ) of Thwaites Glacier, West Antarctica overlaid on (*A*) 2020–2021 ice speed from MEaSUREs and bed elevation from BedMachine Antarctica v3.7 with 100-m bed elevation contour levels (black thin). *Inset* shows glacier location in Antarctica. Mouginot Ridge (MR), South Ridge (SR), and Bull’s Eye (BE) are indicated in black letters. Zoom on (*B*) Thwaites Eastern Ice Shelf (TEIS) overlaid on a Spring 2023 DInSAR interferogram acquired by the RADARSAT Constellation Mission (RCM) and on (*C*) Thwaites Glacier Tongue (TGT) overlaid on a Spring 2024 DInSAR interferogram from the ICEYE constellation, with 100-m bed elevation contours (black thin). The location of the IGZ is in double black solid. (*B*) 2019 Icefin AUV track (solid brown and labeled) and 4 ApRES instruments deployed in January 2020–2021 (white squares labeled 1 to 4). (*B* and *C*) show the seaward boundary of the Ocean Grounding Zone (solid blue. (*A*–*C*) show the flowline used by the MITgcm model (solid yellow).

Over the past forty years, the Antarctic Ice Sheet has contributed rapidly to sea level rise (SLR) and is expected to continue in the coming decades (1). In West Antarctica, glacier retreat has been attributed to warm waters of Circumpolar Deep Water (CDW) origin that reach the glacier grounding line and rapidly melt basal ice (2, 3). Thwaites Glacier contributes one third of the mass loss in West Antarctica (4). The glacier discharges 15% of West Antarctica in area (1, 5) and contains an ice volume equivalent to a global increase in sea level of 0.6 m (6). Thwaites is 120 km wide at its grounding line, with a 20 km wide “main trunk” on the west side that flows at 2.6 kilometer per year (km/y) into the Thwaites Glacier Tongue (TGT) and a 100 km wide segment on the east side that flows at 400 m/y into the Thwaites Eastern Ice Shelf (TEIS) (Fig. 1). The bed topography upstream of the grounding line of TGT is mostly retrograde, that is, the bed elevation deepens inland, which makes the glacier retreat potentially irreversible and a significant source of concern for future sea level rise from Antarctica (7–9). The bed topography upstream of the TEIS is shallower and bumpier, therefore, potentially providing more stability to the glacier.

The grounding line of the TGT has been retreating at 1 km/y from 1996 to 2011 along the main trunk, which is one of the highest retreat rates in Antarctica. The TEIS grounding line along the “butterfly extension” has retreated more slowly and sporadically (10). In recent years, the retreat rate has decreased to 0.5 km/y for TGT, because the grounding line has reached a bump in the bed, herein named the “Mouginot Ridge,” which induces temporary stability of the glacier, until the grounding line resumes its retreat farther inland on deeper beds (11).

High-resolution differential Synthetic Aperture Radar (SAR) interferograms (DInSAR) of Thwaites Glacier acquired by the ICEYE constellation in Spring 2023 have shown that the grounding line, which is the boundary that separates grounded ice from floating ice, migrates many kilometers during the tidal cycle, thus defining a multiple kilometers-wide “Ice Grounding Zone” (IGZ) (11). The IGZ is bounded landward by ice that remains grounded at all tidal stages and seaward by the most advanced position reached by the grounding line at low tide. Seawater intrusions occur within the IGZ at tidal frequencies. Downstream of the IGZ is the so-called “Ocean Grounding Zone” (OGZ), which is always underlaid by seawater and undergoes tidal flexure. Tidal flexure and ice bending affect both the OGZ and the IGZ. Along the main trunk of Thwaites, the length of the IGZ has increased from 2 to 3 km in 2018–2019 (10) to 2 to 6 km in 2023 (11) as the IGZ retreated inland. Occasionally, seawater intrusions extended another 6 km upstream of the IGZ to form a bull’s eye pattern of vertical motion centered in a bed depression upstream of “Mouginot Ridge.” On 10-12 March 2024, ICEYE data revealed that the IGZ and bull’s eye region became fully connected to form a 12-km-long IGZ (Fig. 1*C*).

This kilometer-long IGZ challenges the traditional view of a fixed grounding line with zero melt used in most ice sheet models that project the SLR (12). A long IGZ implies that vertical ice displacements extend far inland, allowing faster, cyclic transport of pressurized, warm, salty water beneath grounded ice, which can melt basal ice in contact with warm ocean waters. High basal ice melt rates will eventually permanently lift the ice off the seabed, thus reducing the basal resistance to flow, making glaciers more prone to acceleration in response to a warmer ocean, increasing the loss of ice mass from Antarctica (13). The impact of ice–ocean interactions on melt rates in the IGZ will depend on the efficiency with which the ocean heat is delivered to the basal ice within the IGZ cavity. The basal melt processes that occur within the IGZ have never been observed in much detail.

Here, we investigate the magnitude of ice melt in the IGZ cavity using a high-resolution ocean model with full physics. We employ a two-dimensional (2D) configuration of the Massachusetts Institute of Technology global circulation model (MITgcm) ocean model along a flow line of the main trunk of Thwaites, or TGT, and along a flow line of TEIS that follows the 2019 Icefin Automated Underwater Vehicle (AUV) pathway (Fig. 1*B*). On the ice-shelf, the model boundaries (ice surface, ice thickness, bathymetry) use the glacier configuration in year 2014 from BedMachine Antarctica v3.7 (8). Within the IGZ, we keep the depth of the bed constant and the thickness of the water column at approximately 1 m with spatiotemporal variations induced by tidal forcing and ice melting. The two configurations of the model are forced with the same tidal forcing. The only difference between them lies in bathymetry and basal ice topography. We prescribe tidal flexing of ice and simulate regular intrusions and extrusions of seawater in the IGZ at tidal frequencies (*Materials and Methods*). We compare the modeled melt rates with observations by the Icefin AUV and four Automated phase-sensitive Radar Echo Sounders (ApRES) deployed under TEIS. We also compare the results of the MITgcm simulations with satellite remote sensing estimates of ice melt in the IGZ of both TEIS and TGT.

## Results

**Fig. 2.**
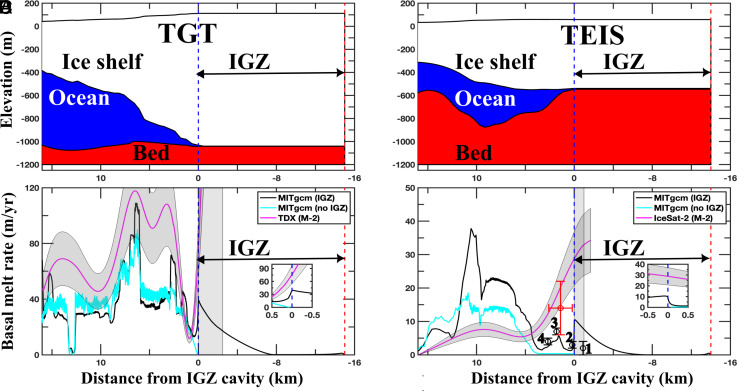
Observed basal melt rates of Thwaites Glacier versus modeled with MITgcm. (*A*) 2D modeling domain for the Thwaites Glacier Tongue (TGT) from BedMachine Antarctica v3.7 (ice surface, ice bottom, and bathymetry) with a 1-m-high, rectangular, uniform Ice Grounding Zone (IGZ) cavity. (*B*) Modeled, tidally-averaged basal melt rates for TGT without an IGZ (cyan) versus including an IGZ (black) compared with satellite-derived annual basal melt rates (magenta) with ± 1σ uncertainty in dark grey shade. Vertical light-gray shading delineates the domain of viability of the satellite estimates (Materials and Methods); the Inset zooms on details at the IGZ cavity entrance. (*C*) 2D modeling domain for the Thwaites Eastern Ice Shelf (TEIS) using BedMachine Antarctica 3.7 with a 1-m-high, rectangular, uniform IGZ cavity. (*D*) Modeled, tidally averaged basal melt rates for TEIS without an IGZ (cyan) versus including an IGZ (black) versus satellite-derived annual basal melt rates (magenta) with ± 1σ uncertainty in dark gray shade. Four ApRES-derived average melt rates ± 1σ are shown with open black circles labeled 1-4, and one Icefin-derived melt rate ± 1σ at red circle with horizontal/vertical error bars. Vertical light-gray shading delineates the satellite-estimate domain of viability; the inset zooms on details at the IGZ cavity entrance.

The IGZ is represented as a rectangular ocean cavity 15 km along the ice-flow direction, consistent with the widest extent seen in satellite data, 1 m initial height at low tide, and with vertical grid cells that can be partially filled to represent a time variable water-column thickness. We ensure a smooth transition in the basal slope at the entrance of the IGZ cavity to avoid singularities in the ice melt (Fig. 2 *A* and *C*) (14). To “smooth out” this transition, we impose that the curvature at the entrance is proportional to the speed of grounding line migration to the power 2/5 as in refs. 14 and 15. The MITgcm ocean model is run at a resolution of 1 m vertical by 20 m horizontal, with and without an IGZ cavity, to model the circulation of water mass, heat, and salt and calculate the basal ice melt rates from the three-equation approach (16). We used DInSAR data to prescribe the tidal flex of the ice. The limit of flexure is fixed at 10 km on the seaward side of the IGZ entrance. The landward limit migrates back and forth at a constant speed equal to the IGZ length divided by the tidal period. Between these limits, the vertical displacement varies linearly with distance from the landward limit, reaching the tidal amplitude at the seaward limit. In response to ice tidal flexing, seawater moves into and out of the IGZ at tidal frequencies. The height of the tide is calculated using the CATS 2018 tidal model (17).

The modeled ice melt rates averaged over a full tidal cycle are highest at the entrance of the IGZ of TGT (Fig. 2). Indeed, thermal forcing is strongest there and decreases with distance into the IGZ cavity as seawater loses heat to melting and mixes with meltwater. The modeled melt rates for the TEIS are lower. In both cavities, the melt rate decreases with distance in the IGZ cavity to zero before the end of the cavity, thus defining an effective cavity length, “$L_{e}$,” beyond which there is no melt. The absence of melt reflects the fact that there is no mixing between the fresh water and the warm ocean water coming to the end of the cavity. $L_{e}$ is 8 km for TGT and 6 km for TEIS. Outside the IGZ, the modeled melt rates drop rapidly with distance before reaching a secondary peak about 6 km downstream of the IGZ for TGT and 10 km for TEIS. The calculated peak melt rate at the entrance of the IGZ cavity is four times (40 m/y) larger for TGT than for TEIS (10 m/y). Hence, the two ice shelves exhibit different melt rates and effective lengths within the IGZ, and distinct melt patterns seaward of the IGZ.

When we exclude the IGZ cavity from the MITgcm ocean model, that is, when we use a fixed grounding line with no tidal flexing, the modeled melt rates are similar outside the IGZ for TGT and halved for TEIS. More importantly, the melt rates approach zero at the grounding line of TGT and TEIS, as prescribed for a fixed grounding line where by definition no inflow can occur (Fig. 2). The difference in modeled melt rates with and without an IGZ is maximal at the cavity entrance, which is where the water speed is the largest with an IGZ versus zero without an IGZ, and the melt increases with higher water speed. The inclusion of an IGZ produces a fundamental increase in the ice melt rate at the grounding line for the thermal driving range tested.

We compare the model results with the in situ data in TEIS, i.e., temperature and melt rates derived from the Icefin AUV, which surveyed a region 2.6 km in length seaward of the IGZ (18, 19). The model results are within the errors of the Icefin observations, which reported low melt rates for the TEIS despite strong ocean thermal forcing available within meters of the ice base. We find a good agreement (7 to 8 m) when comparing the modeled thermocline depth in the two configurations with the thermocline observed by Icefin (*SI Appendix*, Fig. S10). However, in our simulation, we have a thermal bias of 0.5 ^°^C relative to IceFin near the IGZ. This discrepancy likely contributes to the difference in melt rate between the MITgcm model and the IceFin-derived melt rate estimates. The thermocline position varies by 2 m during a tidal cycle, consistent with the tidal amplitude used in the test runs (*SI Appendix*, Fig. S10*B*). We also compare our data with the melt rates of 4 x ApRES stations (Fig. 2*D*). The model results agree well with the ApRES data near the IGZ. The comparison confirms low melt rates in the TEIS IGZ (<10 m/y).

**Fig. 3.**
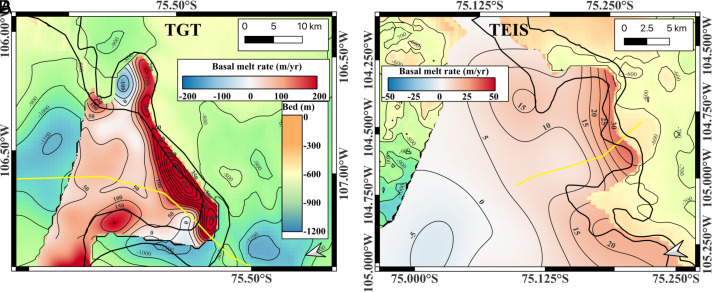
Basal melt rates derived from satellite remote sensing data in years 2021-2022. (*A*) Ice melt calculated using a Lagrangian framework for TGT with TanDEM-X DEMs and MEaSUREs ice velocity. The IGZ Upper and Lower boundaries are thick black lines derived from ICEYE 2024. Contour levels in melt are thin black lines every 50 m/yr. (*B*) Ice melt calculated using a Eulerian framework for TEIS with ICESat-2 ATL14, ATL15 elevation data, and MEaSUREs ice velocity. The IGZ Upper and Lower boundaries are thick black lines derived from RCM 2023. Contour levels in melt are thin black lines every 5 m/yr. In both (*A* and *B*), flow lines used to define the domain of the 2D MITgcm simulations are yellow solid. In areas where we do not calculate melt (grounded ice and outer ice shelf domain), the background is bed elevation from BedMachine Antarctica v3.7 with 100-m contour levels (thin black lines).

In situ data are not available for TGT. However, we compare the MITgcm results with satellite remote sensing estimates of ice melt. For TGT, we calculate the basal melt rates using a time series of TanDEMs-X Digital Elevation Models (DEM) for the years 2021–2022 using a mass conservation approach in a Lagrangian framework (20) (Fig. 3*A*). We also calculate the melt rates with an Eulerian framework using the thickness change products of ICESat-2 ATL15 and the DEM reference ATL14 (*SI Appendix*, Fig. S9*B*). In the case of TEIS, we have no TanDEMs-X DEMs, but we use ICESat-2 data for years 2021–2022 in a Eulerian framework (Fig. 3*B*) (*Materials and Methods*).

The calculation of ice melt from satellite data assumes that ice is in hydrostatic equilibrium (HE). When we compare ice surface elevation and thickness data collected by NASA Operation IceBridge between 2011 and 2018 near the IGZ, we find that the ice deviates from HE by several meters near the IGZ, which introduces uncertainties of several 10 m’s in ice thickness. The bending stresses acting on the grounding line deflect the ice surface below flotation by several meters for a few km downstream of the IGZ. Further downstream, the ice surface lifts above HE by several meters over a region a few km wide (*SI Appendix*, Fig. S1). This deflection is a permanent feature of the ice shelf in addition to short-term fluctuations in surface elevation (±1 m) caused by changes in sea surface height (oceanic tide and atmospheric pressure). In TGT, the deviation from flotation is considerable as it reaches 10 m in surface elevation or 100 m in ice thickness (*SI Appendix*, Fig. S1*B*). The deviation is also large in the TEIS (*SI Appendix*, Fig. S1*D*). As ice travels through the bending zone with one year, its surface first drops below flotation, creating the illusion of rapid melt, then rises in elevation, suggesting freeze-on. We correct for this effect by smoothing the calculated melt rates over the width of the bending zone, i.e., 4 km for TGT and 6 km for TEIS (*SI Appendix*, Fig. S1). We validate this approach by comparing the results with a partial residual map generated with multiyear OIB data (*SI Appendix*, Fig. S2 and S3). We obtain similar values between the Eulerian or Lagrangian approaches (*SI Appendix*, Fig. S9). Our melt rates outside the IGZ are consistent with those in refs. 10 and 21.

**Fig. 4.**
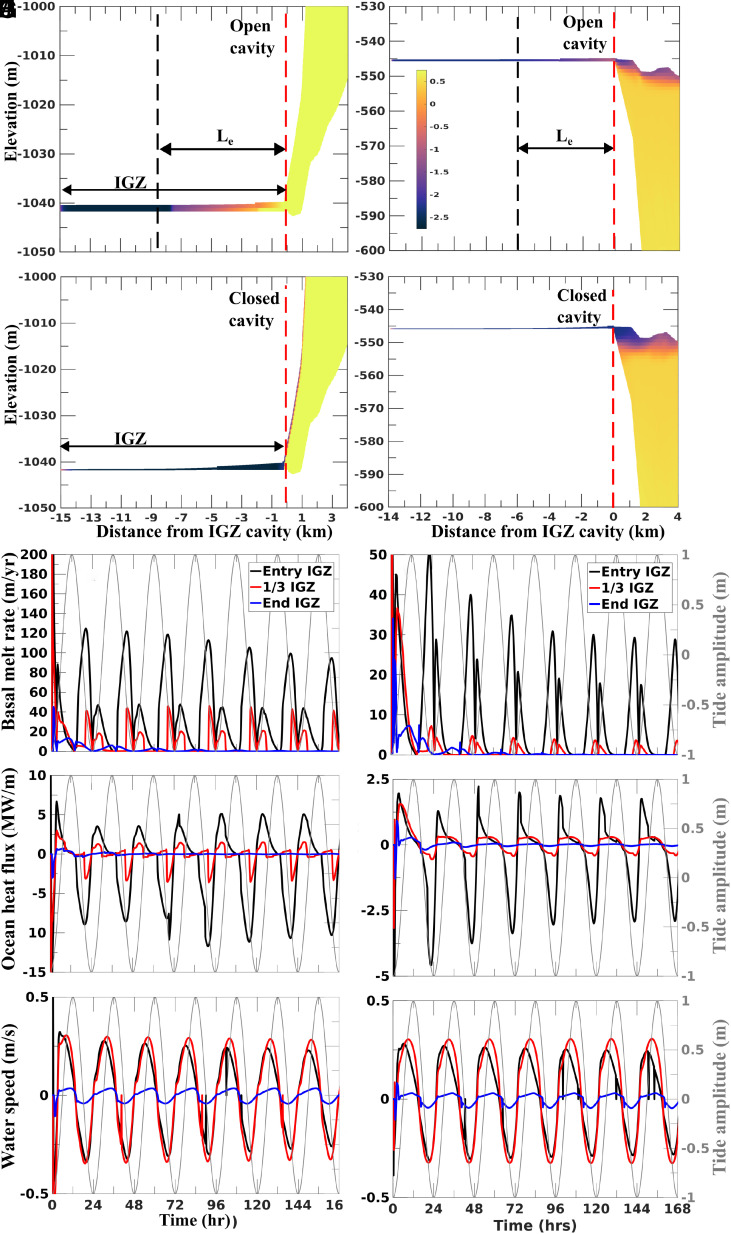
MITgcm modeling of ice-ocean interaction in the IGZ of Thwaites Glacier. (*A*) Temperature in the open cavity configuration for Thwaites Glacier Tongue (TGT), zoomed on the IGZ, with the red dashed line (not a dynamic boundary) indicating the IGZ entrance. Le denotes the maximum effective distance over which melt is active. (*B*) Temperature in the closed cavity configuration for TGT, zoomed on the IGZ, with the red dashed line indicating the IGZ entrance. (*C*) Basal melt rate for TGT at the IGZ entrance (black), one-third into the IGZ cavity (red), and at the cavity end (blue), along with the tidal amplitude (gray). (*D*) Ocean fheat flux for TGT at the same three IGZ positions as in (*C*) and (*D*). Negative values indicate incoming ocean heat flux; positive values indicate ocean heat exiting the cavity. (*E*) Water speed for TGT at the same three IGZ positions as in (*C*) and (*D*). Negative values indicate incoming seawater; positive values indicate outflowing seawater. (*F*) Temperature in the open cavity configuration for Thwaites Eastern Ice Shelf (TEIS), zoomed on the IGZ, with the red dashed line indicating the IGZ entrance. (*G*) Temperature in the closed cavity configuration for TEIS, zoomed on the IGZ, with the red dashed line indicating the IGZ entrance. (*H*) Basal melt rate for TEIS at the IGZ entrance (black), one-third into the IGZ cavity (red), and at the cavity end (blue), along with the tidal amplitude (gray). (*I*) Ocean flux for TEIS at the same three IGZ positions as in (*H*). (*J*) Water speed for TEIS at the same three IGZ positions as in (*H*) and (*I*).

In TGT, the modeled melt rates follow a trend similar to that in the remote sensing data, i.e., a large melt value at the entrance of the IGZ and two peaks in the OGZ. However, the modeled melt rates are lower than the satellite-derived estimates, especially within the IGZ. We address this mismatch later in the discussion. The exchange of ocean heat with basal ice penetrates only half of the IGZ cavity (Fig. 4 *A* and *B*). The model predicts that melt water and seawater stop mixing inside the IGZ at low tide (Fig. 4*A*, *B*, *F*, and *G*) for TGT and TEIS (*Materials and Methods*), especially TEIS. The deeper part of the cavity is less efficient in allowing ocean water to circulate because the intrusions lose momentum (Fig. 4 *E* and *J*) and heat (Fig. 4 *D* and *I*) by the time they reach the end of the cavity. The modeled heat flux at the entrance of the IGZ cavity is 146% higher for the incoming flux compared to the outgoing flux due to the heat loss of the ice melt (Fig. 4*E*). Only 30 to 35% of the incoming heat flux is used to melt the ice. The remaining heat exits the cavity (*SI Appendix*, Fig. S4*A*).

In TEIS, there is good agreement with remote sensing estimates (Fig. 2*D*). Downstream of the IGZ, the model predicts slightly higher melt rates than observed. However, remote sensing estimates confirm that the IGZ melt rates for TGT are much larger than those for TEIS. The melt rates derived from ApRES and the melt rates derived from Icefin (19) are similar to those predicted by MITgcm (Fig. 2*D*). For TEIS, we also find that 30 to 35% of the incoming heat flux is used to melt the ice (*SI Appendix*, Fig. S4*B*).

In both TGT and TEIS, the MITgcm results reveal an asymmetry in the melt during the tidal cycle (Fig. 4 *C* and *H*). However, the tidally averaged and IGZ-length averaged melt values converge to steady values after 5 to 6 tidal cycles. In TGT, the velocity of the buoyant meltwater plume increases rapidly (30 cm/s) outside the IGZ along the steep basal slopes of the OGZ (Fig. 4 *D* and *I* and *SI Appendix*, Fig. S5 *A* and *B*).

The ocean model indicates that the melt rate increases with the length of the IGZ, *L*, and the magnitude of Thermal Forcing, *TF*. A longer IGZ increases the travel speed of seawater as water needs to travel a longer distance at tidal frequencies, hence more heat exchange with the ice and more ice melt. Higher thermal forcing increases heat exchange with ice. We perform numerical experiments with *L* ranging from 5 to 15 km in 1-km steps and *TF* ranging from 2.6 °C to 4.6 °C in steps of 0.5 °C for TGT (*SI Appendix*, Fig. S6). We employ a least-square fit of the simulated average melt rate, $\dot{m}$, and the integrated melt within the IGZ, $\dot{M}$, as $\dot{m}$ = $AL^{b}{\left(TF\right)}^{c}$, where *A* is a constant, *TF* is the thermal forcing of the ocean at a depth of the IGZ, *L* is the length of the IGZ, and *b* and *c* are constants. For $\dot{M}$, we find A = 0.072, b = 1.03, and c = 0.92. For $\dot{m}$, we have A = 4.865, b = 0.4, and c = 0.916. Therefore, the melt increases linearly with *TF* and sublinearly with *L*.

For L=15 km, the flow direction of the seawater changes with the tidal state in the first 750 m of the IGZ cavity. At 1 km from the IGZ, the plume starts to ascend, with a speed modulated by tidal forcing (*SI Appendix*, Fig. S5 *C*–*E*), which generates a transition zone at 750 m. The location of the transition zone changes more rapidly with *L* than with *TF*. For example, it migrates from 750 to 250 m when the IGZ is reduced from 15 to 5 km, while an increase in *TF* from 3.75 to 4.75 °C moves it back by 100 m. As the plume ascends along the base of the ice shelf, it entrains ambient seawater that contributes to more ice melt (*SI Appendix*, Fig. S5 *C* and *D*).

## Discussion

We attribute the differences in melt magnitudes and spatial patterns of TGT and TEIS to their specific geometry and depth of the ocean cavities. The TGT is 1,100 m deep versus 600 m for TEIS; therefore, *TF* is 0.3 ^°^C higher on the TGT from pressure alone. The base of the ice-shelf in TGT is steeper than in TEIS (Fig. 2). A flatter base limits the entrainment speed of the meltwater plume and yields lower melt rates (18, 19). Steep basal ice slopes below the TGT increase the speed of the mixed layer, produce more heat exchange with the ice, and more melt. Previous studies have suggested that the melt rates on the TEIS are too low to explain the fast glacier retreat (18, 19). Here, we find that the unexplored TGT experiences a melt four times higher than that of the TEIS, which is more consistent with the rapid retreat of the TGT in recent decades.

Although the inclusion of a tidally forced IGZ in the model helps elevate melt rates near the IGZ in closer agreement with observations, this process alone cannot fully explain the enhanced melt rates seen in the satellite data. We attribute these differences to several factors. First, MITgcm does not fully resolve the turbulent flow in the IGZ as observed by IceFin (18), potentially underestimating the melt by missing additional heat exchange associated with unresolved turbulence. Second, we use a simplified geometry of the IGZ cavity given the absence of detailed observations. Third, we smooth the basal ice profile in the model to remove rapid variations in the basal slope that can create spurious peaks in the melt (14). Fourth, the spatial resolution of the model is higher than the spacing of the satellite grid, which affects the comparison between estimates. Fifth, the model does not include a layer of subglacial freshwater in the cavity, which may affect the melt regime (22). Subglacial water may oppose seawater intrusion or enhance the thermohaline circulation of the cavity; hence, lower or increase the melt rates. Sixth, the 2D ocean model configuration prevents meltwater from escaping the cavity in a direction perpendicular to the 2D domain. This limitation will lower the melt rates, as found in the case of a melt water plume rising along a 3D vertical calving face (23).

A 3D geometry will allow seawater to follow more complex pathways beneath the ice shelf. In prior studies of TGT, seawater has been shown to first reach the eastern edge of the domain, where melt rates are high, and circulate clockwise around the grounding line toward the western edge, where melt rates are low (2). In the presence of an IGZ, we expect a similar circulation, with enhanced melting at the eastern edge due to the rising meltwater plume, tidal pressure gradients, and the Coriolis force. Additionally, at the glacier edges, the ice flexure is three-dimensional. To minimize 3D effects, we used a center flowline away from regions of high melting or refreezing.

The buoyant meltwater moves at high speeds along the slopes of the ice-bottom cavity of the TGT, thus establishing an efficient circulation of CDW in the cavity, causing higher melt rates (Fig. 4 *D* and *I* and *SI Appendix*, Fig. S5 *A* and *B*). The water speed is lower for TEIS. In the TEIS, the fresh and cold melt water at the top of the cavity acts as an insulating layer that protects the ice from warm, salty ambient seawater (Fig. 4 *F* and *G*), leading to the low melt rate (Fig. 4*H*). The melt rate is conditioned by the stratification of the water outside the cavity (24, 25) and the shape of the cavity. Previous studies have shown that melt rates below the TEIS are low because vertical shear is not strong enough to overcome stable water stratification (19).

In TEIS, the thermocline depth averaged over the first 3 km outside the IGZ cavity is slightly deeper in the model (Fig. 4 *F* and *G*) than observed by Icefin (*SI Appendix*, Fig. S10). We attribute it to two factors: 1) the model uses a smoothed version of the ice base that does not include the high-frequency undulations observed by Icefin that improve localized vertical mixing, resulting in a more mixed water column, with higher temperatures near the ice base and cooler temperatures in the upper 10 m of the ice base (50 to 75 m). 2) the three-equation melt parameterization used by the model overestimates the melt rates in regions of weak vertical shear, leading to a colder, fresher, and more buoyant melt plume than observed (*SI Appendix*, Fig. S10*B*).

The thermal bias of 0.5 ^°^C in the MITgcm ocean model arises from limitations in the cavity geometry and the evolution of the water mass in the model. Specifically, 1) we initialize ocean properties many 10km downstream of the IGZ using one CTD, 2) the full 3D geometry of the cavity is poorly constrained by observations, 3) the model employs a smoothed 2D cross-section with a simplified 40 km ice-shelf length; our results show that the slope of the ice-shelf base strongly controls both melt magnitude and spatial distribution. This bias is reasonable in the context of our study, which focuses on capturing the spatial melt pattern, isolating, and assessing the first-order impact of including an IGZ representation, rather than an exact pointwise agreement with sparse data.

The melt asymmetry predicted by the model is consistent with pressurized, warm, intruding seawater that produces more melt than cooler, fresher outgoing seawater (Fig. 4 *C* and *H*), as on the Ross Ice Shelf (26). The outgoing mixture of seawater and meltwater is resistant to flow as it moves against density gradients, which contributes less melt, as in ref. 15.

The melt parameterization in the IGZ for TGT is useful to quantify the impact of seawater intrusions on the ice melt rates given *TF* and *L* The melt intensity will change with different cavity geometries, but the sensitivity to *L* and *TF* should remain the same. Remote sensing observations show that *L* is shorter with steep slopes in the seabed and low basal water pressure, and longer with shallow slopes in the bed and high basal water pressure (11). We attribute the sublinear dependence of the melt rate on *L* to the fact that water has to counteract friction at the ice base and along the sea bed, which makes the cavity less efficient in circulating heat.

The modeled seawater intrusions are longer ($L_{e}$ is 8 km for TGT and 6 km for TEIS) than those predicted by the classical layered seawater intrusion model over flat, hard beds (3.7 km for both TGT and TEIS) (27) (*Materials and Methods*). The MITgcm model also predicts that TGT has higher peak melt rates and longer intrusions than TEIS under identical tidal forcing, which contrasts with the classical theory that predicts equal seawater intrusion lengths for TEIS and TGT. This difference is not due to the higher effective seawater velocities in our simulations (30 cm/s) versus the classical theory (8 cm/s), even including a time-averaged tidal forcing (28); it reflects fundamentally different physical processes at play. The MITgcm model incorporates the tidal flexing of ice, which pumps seawater into and out of the IGZ cavity at each tidal cycle versus the classical theory, which only represents the diffusion of waters of differing densities. The tidal pumping mechanism is more efficient in transporting seawater than the diffusion process, as demonstrated by our simulations.

The layered seawater intrusion theory proposes a linear parameterization of the melt rate, with the melt peak at the IGZ entrance (27). Our modeled melt rates experience a quadratic decay, which we attribute to two factors. First, thermal forcing and water velocity decrease approximately linearly within the IGZ cavity, resulting in a quadratic decrease in melt. Second, the melt asymmetry is less pronounced deeper into the IGZ cavity (40 m/y melt asymmetry at the IGZ entrance versus 20 m/y at one-third of the cavity length), which further contributes to a nonlinear profile.

Our choice of a vertical resolution of 1 m limits our ability to model small-scale turbulence at the ice–ocean interface. Fully resolving these boundary layer processes would require centimeter-scale Large Eddy Simulations (LES) or Direct Numerical Simulations (DNS) (24, 29). However, extending the LES or DNS frameworks to include the coupled processes of tidal flexure and grounding-line migration remains technically challenging and computationally prohibitive at this time.

Within our MITgcm framework, increasing vertical resolution to 0.3 m by including more vertical cells in the IGZ ocean cavity would not fully resolve these dynamics, as a constant eddy diffusivity scheme still limits entrainment and mixing in the meltwater layer. Although this sensitivity test was performed for a different glacier system, the findings are applicable here due to the similar melt patterns and underlying physical processes. This configuration produces a stratified boundary layer that insulates the ice from the underlying seawater, suppressing the peak melt rates by 10 to 20% compared to the representations of 1-m single cells (14). Importantly, however, we find that this suppression has little influence on tidally averaged melt rates or seawater intrusion distances, suggesting that the bulk exchange remains reasonably captured in our simulations. These findings underscore the importance of exploring more physically based vertical mixing parameterizations before pursuing costly higher-resolution simulations.

Using satellite data and numerical ocean modeling, we find contrasting melt regimes in TGT and TEIS. Satellite analysis, despite higher uncertainties in resolving melt within the IGZ (30), is consistent with the observation of Icefin and ApRES in TEIS. However, the model results and remote sensing estimates indicate higher melt rates in TGT. As TGT carries the dominant fraction (80%) of the mass flux of Thwaites Glacier compared to TEIS, its high melting regime will dominate the evolution of the glacier system. We recommend in situ explorations of the TGT cavity to confirm the model predictions and evaluate the remote sensing estimates in more detail.

The modeling results have implications for modeling the evolution of Thwaites Glacier. Most ice sheet models do not include an IGZ with vigorous melt. Excluding the IGZ underestimates the ice melt at the grounding line, which limits the impact of warm ocean waters on glacier evolution. Including kilometer-scale intrusion in the IGZ will yield higher basal melt rates over many kilometers of grounded ice. As ocean waters become warmer, higher melt rates will be applied over a larger area of basal ice, which may separate from the bed, which will reduce basal resistance to flow and, in turn, allow for greater glacier speeds in response to ocean warming.

Previous ice sheet modeling studies (12, 27, 31, 32), which did not use IGZ observations and high-resolution ocean modeling, have already shown the importance of including km-scale seawater intrusions to predict glacier evolution. For TGT (33), the modeling effort was based on the assumption that seawater intrusions cause a bright ice bed interface in radar sounding echoes beyond the grounding line. Other authors have suggested that layered seawater could flow under a freshwater wedge in the IGZ without tidal pumping (27). Regardless of the mechanism by which seawater intrudes beneath ground ice, these modeling studies agree that seawater intrusions in the kilometer-wide IGZ increase the loss of glacier mass, up by a factor of two (34).

We have observational evidence and ocean modeling results to document that kilometer-scale seawater intrusions beneath grounded ice generate a high melt in the IGZ of TGT. Although seawater intrusions are thin (several 10 cm to 1 m), the ocean model indicates that they are capable of producing high melt rates in favorable IGZ geometries. More advanced studies will require detailed observations of the shape of the cavity, the roughness and frictional properties of the basal ice and seabed interface, the impact of subglacial water outflow on seawater intrusions, the impact of turbulence at the ice–ocean interface in the IGZ, and the magnitude of melt rates (26).

We conclude that observations and ocean models call for a revision of ice sheet models to include kilometer-scale seawater intrusions beneath grounded ice (here 1 to 15 km) (11, 35) that can produce high rates of basal ice melt over a considerable distance (14, 30). This modification will increase the sensitivity of the model to ocean warming and, in turn, will increase the projections of the increase in sea level of Thwaites and other glaciers (27, 33). The same conclusions will apply to Greenland. More work is required to solidify the relationship between IGZ length, bed slope, cavity shape, and basal water pressure.

## Materials and Methods

TanDEM-X DEMs are distributed at 8 m spacing (36) and calibrated with ICESat-2 ATL14 data. We estimate the absolute bias in surface elevation to be ±2 m. We calculate tides and Inverse Barometer Effect (IBE) using an offshore location (106.4°W, 74.7°S), the CATS2008 tidal model, and ERA5’s surface pressure reanalysis data (35). The firn depth correction and geoid correction are from BedMachine v3.7.

ICESat-2 DEMs combine the reference 2020 ATL14 DEM at 100 m post and ATL15 surface elevation change DEMs at 1 km post (37). We use linear time interpolations to extract the annual thickness change at the time stamp of each TDX DEM. We convert the annual surface elevation change into thickness change assuming flotation.

Ice velocity is from European Space Agency (ESA)’s Earth Remote Sensing (ERS) 1 and 2, Envisat ASAR, Sentinel-1 and 2, NASA’s Landsat 7 and 8, Canadian Space Agency (CSA)’s RADARSAT-1, Japanese Space Agency (JAXA)’s ALOS PALSAR, and the United States Geological Survey (USGS)’s Landsat-4/5 (38) available at NSIDC (NSIDC-0754).

Operation ice bridge data OIB includes the Multichannel Coherent Radar Depth Sounder (MCoRDS) ice thickness data and the Airborne Topographic Mapper (ATM) surface elevation data. We fill gaps in ATM elevation (clouds) with the radar-derived surface elevation. We calculate ice thickness from flotation using the ATM data, $H_{\mathrm{ATM}}$ with no firn air correction (FAC). We then select calibration points on the ice shelf (red line in *SI Appendix*, Fig. S7*A* for TGT and *SI Appendix*, Fig. S8*A* for TEIS) to calculate a mean FAC that minimizes the residual *R* = $(H_{\mathrm{ATM}}-H_{\mathrm{MCoRDS}})/9.26$, where $H_{\mathrm{MCoRDS}}$ is the MCoRDS ice thickness. FAC is 7.7 m for TGT (*SI Appendix*, Fig. S7*B*), as in ref. 39 and models (40, 41); and 8.9 m for TEIS (*SI Appendix*, Fig. S8*B*).

Deviation in ice thickness from flotation used to determine the width of the Bending Zone (BZ), is calculated using OIB surface and thickness data and an optimal interpolation approach (42). First, we compute the deviation in thickness for the 2011–2012 OIB tracks (solid green lines in *SI Appendix*, Fig. S2*A*). We apply a Marr wavelet filtering (Gaussian hat functions) with a decorrelation scale of 1 km (track spacing), orienting the wavelet’s major axis perpendicular to the tracks and the minor axis along them, to interpolate the results. This filtering produces a 2D residual map for the year 2011–2012 that fills the domain seaward of the solid blue lines in *SI Appendix*, Fig. S2*A*. We shift this map to align with the 2023 IGZ geometry using a set of control points (*SI Appendix*, Fig. S2*B*). We then incorporate the 2018 OIB residuals as new observations to produce the 2D residual map in *SI Appendix*, Fig. S2*C*.

Ice shelf basal melt rates are derived from TanDEM-X and ICESat-2 DEMs assuming HE and a: 1) Lagrangian and 2) Eulerian frameworks, respectively (20). If the ice surface rises during the tidal cycle, it is assumed to be within 1 m of flotation. Surface mass balance is from RACMOv2.3p2 (0.1 m/y error). The melt value is set at the particle midpoint in the Lagrangian framework. The uncertainty in melt rates combines several sources: 1) Surface elevation errors of 2 m from TanDEM-X translating into an error, $a_{1}$, of 18.5 m in thickness due to DEM baseline errors, surface penetration, and misregistration (36); 2) a 30 m error, $a_{2}$, in converting elevation to thickness based on hydrostatic versus radar-derived thickness (*SI Appendix*, Fig. S1); 1) and 2) combine into an error of 6% in thickness when averaged over time and space for 2017–2021; 3) an error in surface speed, $u_{\mathrm{err}}$ 3 m/y, from the weighted nominal errors in multiyear mosaics (43); 4) a 0.1 m/y or 0.9% error in SMB; and 5) additional sources such as firn correction, density assumptions, and Gaussian smoothing for 1 to 3%. The relative uncertainty in ice flux is $\left(a_{1}+a_{2}\right)/H+u_{\mathrm{err}}/U$, where U 1,000 m/y and H 1,000 m, i.e., 24.1% in thickness, 0.28% in speed, for a total 24.4% in flux. Independent errors in SMB and thickness change up the total to 25.2%. Additional sources yield a melt error of 26 to 28%. Varying the Gaussian filter width in 0.1 km steps within a ±0.5 km range introduces a 2.5% uncertainty in melt rate.

The “domain of viability,” i.e., the region where melt rates can be determined to within the uncertainty described above, extends up to half the annual horizontal ice displacement from the grounding line since we use DEMs separated by one year to calculate melt. Beyond this limit, the ice surface is above flotation.

On TGT, we compare two methods to account for bending stresses. These bending stresses arise due to the flow of an ice slab into the ocean, not the tidally driven bending stress discussed in ref. 44. When using satellite data to calculate the basal melt rate, we use long time scales, hence we do not account for the tidal bending stresses and simply correct the DEMs for the mean tidal height. Method-1 (M-1) uses the OI residual map to correct the TanDEM-X DEMs before calculating the melt rate for 2020–2021. We apply a 1-km wide Gaussian filter (4 *σ* is the bending zone width). Method-2 (M-2) generates the Lagrangian map using the uncorrected TanDEM-X DEM and applies the same 1-km Gaussian filter (*SI Appendix*, Fig. S2 *A* and *B*). The two methods agree to within ±30 to 50 m/y. We compare the ocean model estimates with the M-2 products.

The icefin temperature data varies in time, space, and depth. We assume that these data are collected at a single location and representative of the oceanic conditions near the grounding line. We bin the data vertically into 1 m nonoverlapping bins, i.e., the vertical resolution of the ocean model, to generate the temperature profile in *SI Appendix*, Fig. S10*A*. We extract the modeled temperature profile over the first 0.2 to 2.6 km seaward of the IGZ ocean cavity in the open and closed configurations and average it spatially in the x-direction (*SI Appendix*, Fig. S10*B*). The thermocline depth for both configurations (dotted lines in B) are within 7 to 8 m of the observed thermocline.

The MITgcm ocean model is a finite-volume method that solves the Boussinesq hydrostatic form of the Navier–Stokes equations on an Arakawa C-grid for an incompressible fluid (45). It incorporates the SHELFICE package, which handles the thermodynamic interactions between ice and ocean (46) and calculates the heat and salt fluxes at the ice–ocean interface and melt rates by solving the three-element equations in ref. 16 using a velocity-dependent transfer coefficient (47). We use a vertical remesh package to model time-variable ice-shelf cavities, accounting for both basal melting and tidal deflections of the ice (48).

The model is configured in 2D, with the x-direction aligned with the water flow direction and the z-direction vertical. All prognostic variables, e.g., velocity, salinity, and temperature, are functions of x and z only and are assumed invariant in the cross-flow (y) direction. While the model solves the full 3D momentum equations, this symmetry assumption effectively reduces the problem to 2D. We neglect the Coriolis force. Although there can be a nonzero velocity in the y-direction, the y-velocity is assumed to be uniform.

The model parameters are listed in *SI Appendix*, Table S1. The domain length is 63 km, with 15 km of IGZ cavity, 30 km of ice shelf, and an open ocean length of 18 km. At the oceanward lateral boundary, we prescribe the salinity and temperature values from year 2018 CTD, closest to the flowlines of Thwaites Glacier (49). In the far field, we impose a “relaxation boundary condition” where we restore the water properties to the CTD profile with a relaxation scale of 1 d within a region extending 5 km from the boundary. The results in the IGZ are not sensitive to the choices of this relaxation boundary because it is far from it. Each experiment is initialized with a horizontally homogeneous temperature-salinity profile in the entire domain. The parameterization for the ocean heat flux, melt rate, and basal drag within the IGZ is the same as for the ice shelf.

The MITgcm model must conserve water volume, i.e., the water column must adjust to tidal flexing. When tidal loading causes the grounding line to migrate landward, the floating ice seaward of the grounding line rises, creating a void. Water from the IGZ cavity flows out to fill this void. Conversely, as the tidal height decreases and the grounding line moves seaward, the ice shelf displaces water, which flows back into the IGZ cavity. This mechanism results in seawater intrusions and extrusions that occur 180^°^ out of phase with the tide, with a maximum cavity height of 1 m, and a mean of 0.5 m. We prescribe a minimum cell height *ϵ* of 10 cm, which is the IGZ water height in the closed configuration. This threshold is analogous to a permanent layer of subglacial water beneath the grounded ice produced by basal friction and geothermal heating.

Within the effective cavity length $L_{e}$, basal melting increases the water column thickness. For TGT, by the end of six tidal cycles, melting adds 0.6 m of water column height at the IGZ entrance versus 0.2 m on TEIS. This change produces different water column thicknesses in TGT and TEIS, however, it only changes the ice basal slope to second order (0.6 m in 7.5 km on TGT and 0.2 m in 6 km on TEIS), which is negligible compared to the spatially averaged slopes (400 m over 15 km on TGT and 120 m over 15 km on TEIS). When the water column is thicker than 1.12 m (1.12% of the vertical resolution), the IGZ is represented with 2 vertical levels, with the original vertical level retaining 1 m thickness.

We do not force any cells in the model to be filled with meltwater. We use the model temperature to identify cells where the thermal forcing, *TF*, is zero, i.e., melt water cells. Cells landward of $L_{e}$ are filled with melt water. Freezing is allowed, but *TF* does not drop below 0. We perform numerical experiments to assess the model sensitivity to viscosity, diffusivity, bed drag, and basal drag coefficients (*SI Appendix*, Table S2). The modeled melt rates change by less than 5% when we change these parameters by one order of magnitude.

Incoming heat flux for melting ($H_{\text{in}}$) is from MITgcm-resolved ocean velocity and temperature fields without averaging (*SI Appendix*, Table S1). The model calculates spatially varying heat flux at the ice–ocean interface, which is integrated along the IGZ to estimate melt-driving heat flux ($H_{\text{melt}}$); negative values indicate inflow and positive values indicate outflow (*SI Appendix*, Fig. S4).

The tidally driven flexure and grounding line migration are introduced in the MITgcm model by prescribing a change in ice-shelf mass calculated to match the desired vertical motion of the ice base (14). Tidal forcing is a diurnal tide with an amplitude of ±1m based on the CATS2008 model averaged over four years. The spatial pattern of ice flexure is as follows: The vertical displacement is zero at and landward of the GL; between the GL and the OGZ, the vertical motion of the ice increases linearly with distance to reach the tidal height and remains constant seaward of the OGZ. We prescribe ice flexure in the IGZ and OGZ, as well as the flushing of seawater in and out of the IGZ (*SI Appendix*, Fig. S11). In response to tidal loading, the grounding line is forced to migrate back and forth, with a speed equal to the IGZ length divided by half of a tidal cycle.

The main difference between observed and modeled tidal flexure is the omission of landward-propagating flexural waves and bed deflection landward of the grounding line, as in refs. 15, 31, and 32. These effects are localized and secondary. Ice melt scales with water speed, which depends on the pressure gradient. The gradient introduced by flexural waves is the vertical deflection (mm) over the wavelength (km), or $10^{-6}$, which is negligible. We also neglect 3D bending near ice edges or pinning points and assume a constant migration speed, consistent with ref. 50.

The seawater intrusion distance is computed using the layered seawater intrusion theory with hard, flat beds (27). This theory describes a flow of freshwater with velocity $U_{0}$ over seawater at rest until a steady-state configuration is reached, where freshwater forms a wedge over seawater. The original theory excludes tidal forcing. Following ref. 28, we select $U_{0}$ as the time-averaged absolute speed of meltwater pulses to represent tidal effects. We idealize the tidal cycle as comprising two distinct phases: a half-cycle of seawater intrusion with negligible meltwater outflow and a half-cycle of meltwater outflow in which the velocity follows a half-sine profile with amplitude $v_{a}=0.25ms^{-1}$ at the GL (black solid line in Fig. 4*E*). This simplification enables the computation of the time-averaged freshwater velocity $U_{0}$ in the intrusion model. In reality, meltwater motion occurs throughout the tidal cycle and the transition between phases is gradual rather than abrupt. The time-averaged speed is $2/\pi \times v_{a}$, which is 0.08 m/s for both TGT and TEIS. We assume a drag coefficient between the water and bed of $1.5\times 10^{-3}$, consistent with MITgcm, a mean cavity height ($H_{0}$) of 0.5 m (the mean cavity height over a tidal cycle, a reduced gravity g’ = 0.28 m^2^/s and a seawater density of 1,028 kg/m^3^ to calculate the depth-based Froude number and deduce an upper limit of seawater intrusion distance to be 3.7 km. This value is the same for TGT and TEIS because the formula depends on $U_{0}$ and $C_{d}$, which are the same for both ice shelves.

A three-equation melt parameterization represents the thermodynamic interactions between the ice shelf and the ocean (16). Two equations are the conservation of heat and salt in the boundary layer. The third equation expresses the relationship of the salinity-dependent, pressure-dependent freezing temperature of seawater with parameters a,b,c:

[1] $$\begin{array}{c} {Q_{I}}^{T}+{Q_{M}}^{T}=-L_{f}\rho_{M}\dot{m} \end{array}$$

[2] $$\begin{array}{c} {Q_{I}}^{S}+{Q_{M}}^{S}=-\rho_{M}\dot{m}S_{B} \end{array}$$

[3] $$\begin{array}{c} T_{B}=aS_{B}+b+cp_{B}, \end{array}$$

where ${Q_{I}}^{T}$ and ${Q_{I}}^{S}$ are conductive heat and diffusive salt fluxes through the ice-shelf, ${Q_{M}}^{T}$ and ${Q_{M}}^{S}$ are diffusive heat and salt fluxes through the ice-shelf/ocean boundary layer, $L_{f}$ is the latent heat of fusion, $\rho_{M}$ is the ocean mixed layer density, $T_{B}$ and $S_{B}$ are the temperature and salinity of ice-shelf base, $p_{B}$ is the pressure at the ice-shelf base, and $\dot{m}$ is the melt rate. The diffusive salt flux on the ice shelf is neglected, so ${Q_{I}}^{S}=0$. The conductive heat flux is the following.

[4] $$\begin{array}{c} {Q_{I}}^{T}=\rho_{I}c_{\mathrm{pI}}\kappa \frac{\left(T_{\mathrm{ice}}-T_{B}\right)}{D}, \end{array}$$

where $\rho_{I}$ is the density of the ice, *κ* is ice-shelf thermal conductivity, $T_{\mathrm{ice}}$ is the temperature of the ice and *D* is the thickness of the ice-shelf. Similarly, we have

[5] $$\begin{array}{c} {Q_{M}}^{T}=\rho_{M}c_{\mathrm{pM}}^{T}\gamma_{T}\left(T_{M}-T_{B}\right);{Q_{M}}^{S}=\rho_{M}c_{\mathrm{pM}}^{S}\gamma_{S}\left(S_{M}-S_{B}\right); \end{array}$$

where $c_{\mathrm{pM}}^{T}$ is the heat capacity of the mixed layer, $c_{\mathrm{pM}}^{S}=1$, $\gamma_{T,S}$ are the turbulent heat and salt exchange velocities calculated using

[6] $$\begin{array}{c} \gamma_{T,S}=\Gamma_{T,S}\sqrt{C_{d}}U_{M}, \end{array}$$

where $C_{d}$ is a dimensionless drag coefficient, $\Gamma_{T}$ and $\Gamma_{S}$ are turbulent heat and salt transfer coefficients, and $U_{M}$ is the water velocity in the boundary layer. The three equations are solved for 3 unknowns: $T_{B}$, $S_{B}$, and $\dot{m}$.

## Supplementary Material

Appendix 01 (PDF)

## Data Availability

Code and data have been deposited in MITgcm code https://doi.org/10.5281/zenodo.8208482 (51), MITgcm modified code https://doi.org/10.5281/zenodo.8250817 (52), Python code for ICESAt-2, TDX https://doi.org/10.5281/zenodo.12658529 (53), https://doi.org/10.5281/zenodo.12785929 (54), and https://doi.org/10.5281/zenodo.12659151 (55), Code for comparing MITgcm and observations https://doi.org/10.5281/zenodo.12659245 (56), and Model output products https://doi.org/10.5281/zenodo.12667971 (57).
