# Supplementary material for: Contrasting melt regime in the Ice Grounding Zone of Thwaites Glacier, West Antarctica
Source: Proc Natl Acad Sci U S A. 2025 Nov 17;122(48):e2512626122. doi: 10.1073/pnas.2512626122 (PMC12685041; doi:10.1073/pnas.2512626122)
Supplement: Supplementary file 1 — Appendix 01 (PDF) [file pnas.2512626122.sapp.pdf]

Supporting Information for

Contrasting melt regime in the Ice Grounding Zone of Thwaites Glacier, West Antarctica

Ratnakar Gadi, Eric Rignot, Dimitris Menemenlis and Bernd Scheuchl

Eric Rignot.
E-mail: erignot@uci.edu

This PDF file includes:

Figs. S1 to S11
Tables S1 to S2

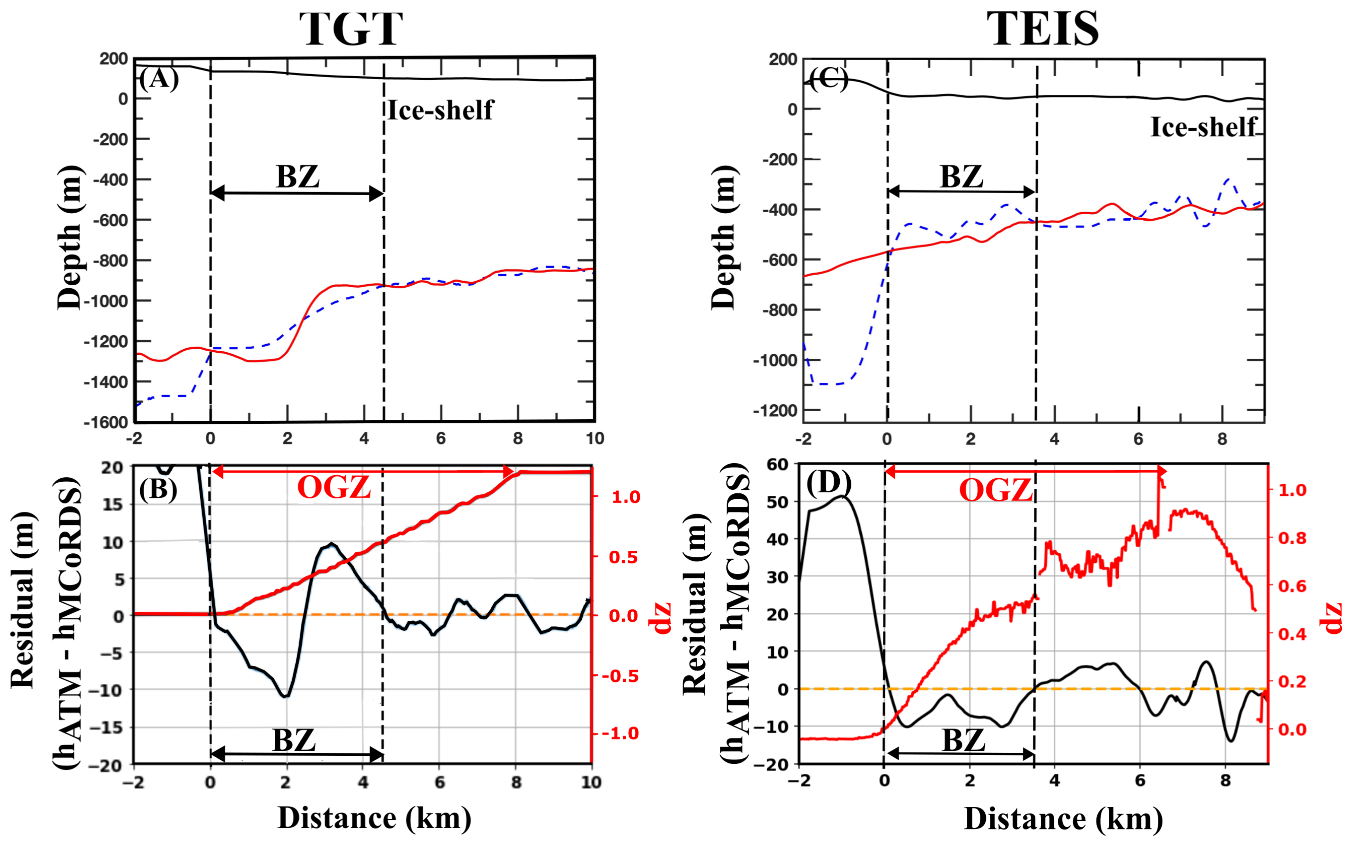

Figure. S1. Determination of the Bending Zone (BZ) of Thwaites Glacier from ATM laser altimetry and MCoRDS ice-sounding radar data. (A) 2D cross-section of TGT with ATM surface elevation (black solid), ATM-derived thickness (blue dotted), and basal elevation from ice-sounding MCoRDS radar data (red solid) acquired on Nov. 15, 2018. (B) ATM minus MCoRDS-derived surface elevation assuming flotation using a 450-m averaging window (solid black) and tidal flexure derived from a 2019-2020 CSK DInSAR interferogram (solid red) versus distance from the 2020 GL. In the BZ, moving from grounded to floating ice, the residual drops to negative (i.e., ice below flotation), then positive (i.e., ice above flotation), and zero again (full hydrostatic equilibrium). (C) 2D cross-section of TEIS with ATM and MCoRDS data acquired on Nov. 21, 2013. (D) Residual surface elevation and tidal flexure derived from an April 4, 2011 CSK DInSAR interferogram. **In (D), we do not see the same residual pattern as in (B) because the ATM and MCoRDS track crosses the IGZ twice instead of only once in the main trunk.**

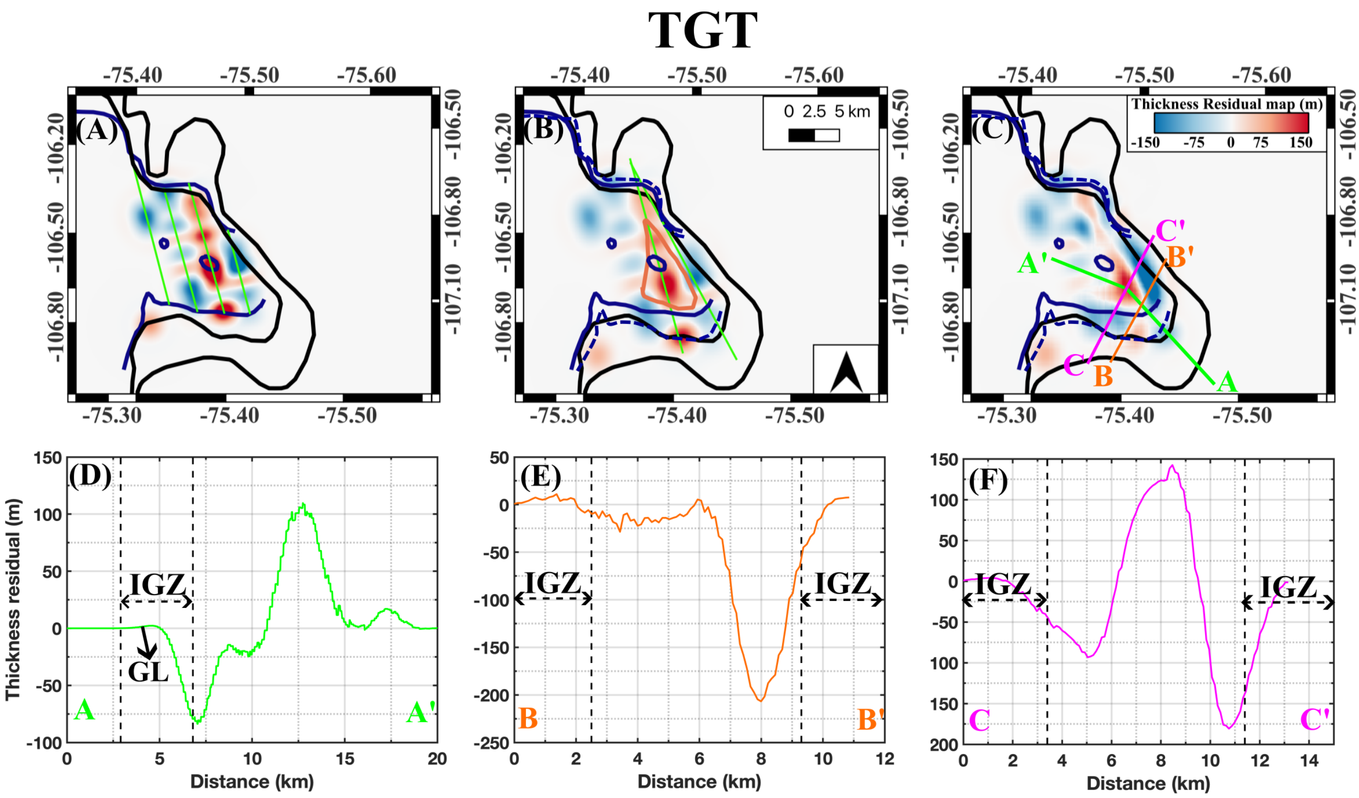

Figure. S2. Thickness residual derived from ATM laser altimetry minus measured from ice-sounding MCoRDS radar using Optimal Interpolation (OI) for Thwaites Glacier Tongue (TGT). (A) MCoRDS and ATM tracks from years 2011 and 2012 (solid green lines), ICEYE 2023 IGZ (black solid lines), and ERS-1 2011 GL (solid blue lines). (B) Thickness residual in (A) georeferenced to the ICEYE 2023 IGZ using a transformation also applied to the ERS-1 2011 GL line (dashed blue line), two additional ATM/MCoRDS tracks from 2018 (solid green), and locations selected for forcing thickness residual to zero (i.e., ice in HE) outside the 2023 flexure zone using a Spring 2023 ICEYE differential interferogram (solid orange) (interferogram not shown). (C) final thickness residual in (B) with the assimilation of the 2018 ATM laser MCoRDS ice-sounding radar data and of the zone of zero thickness residual, and with profile lines A–A′, B–B′, and C–C′ shown in green, orange, and pink, respectively. (D–F) Thickness residuals along the profile lines from (C) with labeled IGZ locations.

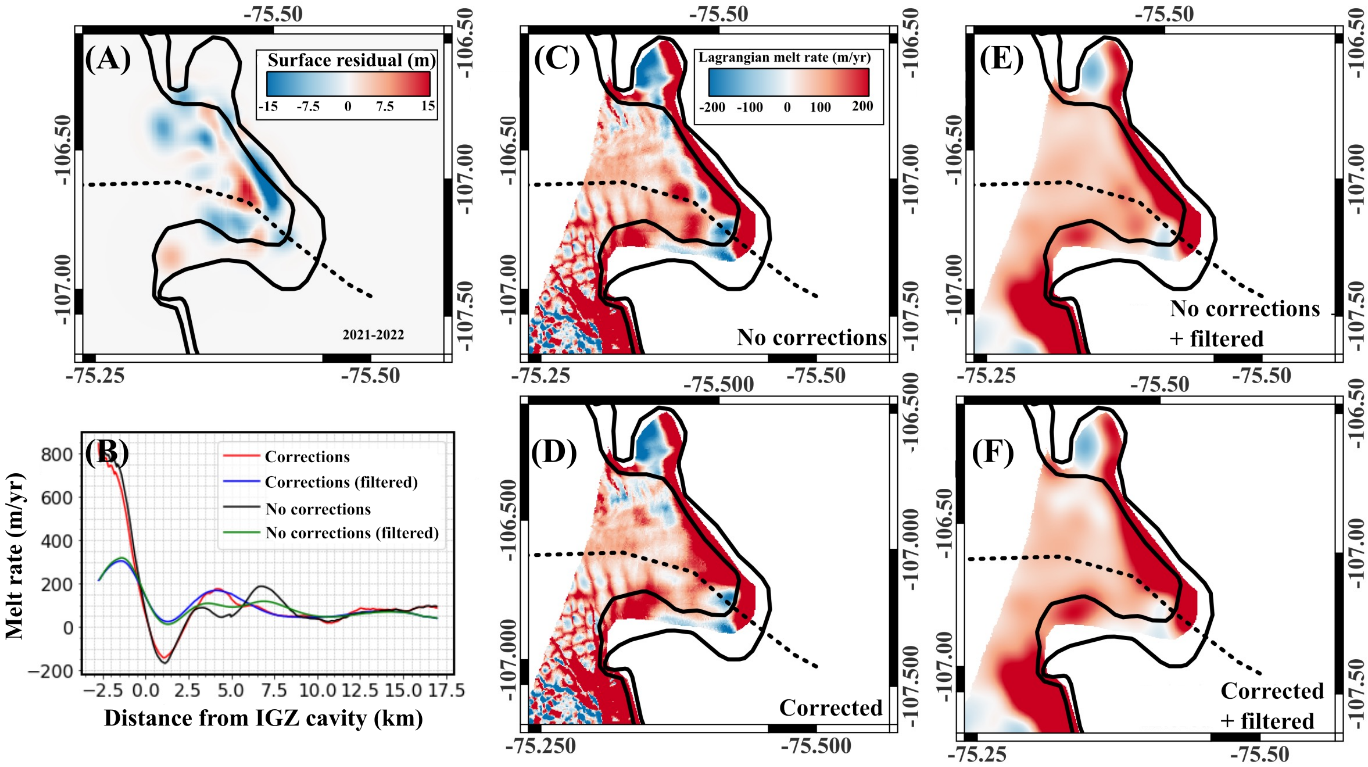

Figure. S3. Impact of time-independent bending stresses on the estimation of ice melt rates for Thwaites Glacier Tongue (TGT). (A) Optimally Interpolated (OI) residual map deduced from ATM/MCoRDS data in 2011, 2012, and 2018 with ICEYE 2023 IGZ (black solid) and flow line used by MITgcm (black dashed line). (B) Lagrangian melt rates along the flow line with and without correction for bending stresses (black and red, respectively) and Gaussian-smoothed solution (green and blue, respectively); (C-D) Lagrangian melt rate for 2021-2022 using TanDEM-X DEM data with C) no correction and D) correction for bending stresses. (E-F) same as (C-D) after Gaussian filtering of 1 km, i.e. 4 x 1 σ matches a 4-km long bending zone.

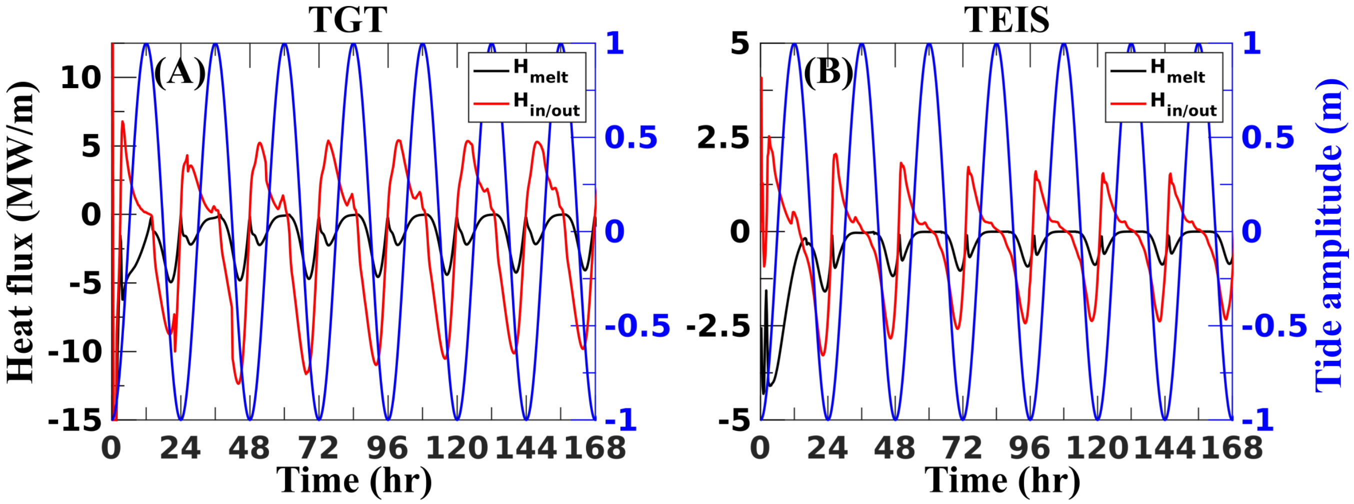

Figure. S4. Modeled ocean heat flux in the IGZ of Thwaites Glacier. Heat flux, H_{in}, in 10^6 Watt/meter (MW/m) entering **(negative)** and leaving **(positive)** the IGZ cavity (red) versus heat flux, H_{melt}, used for melting ice (black) and tidal amplitude (blue) for (A) TGT and (B) TEIS as a function of time (hour, hr) for several tidal cycles.

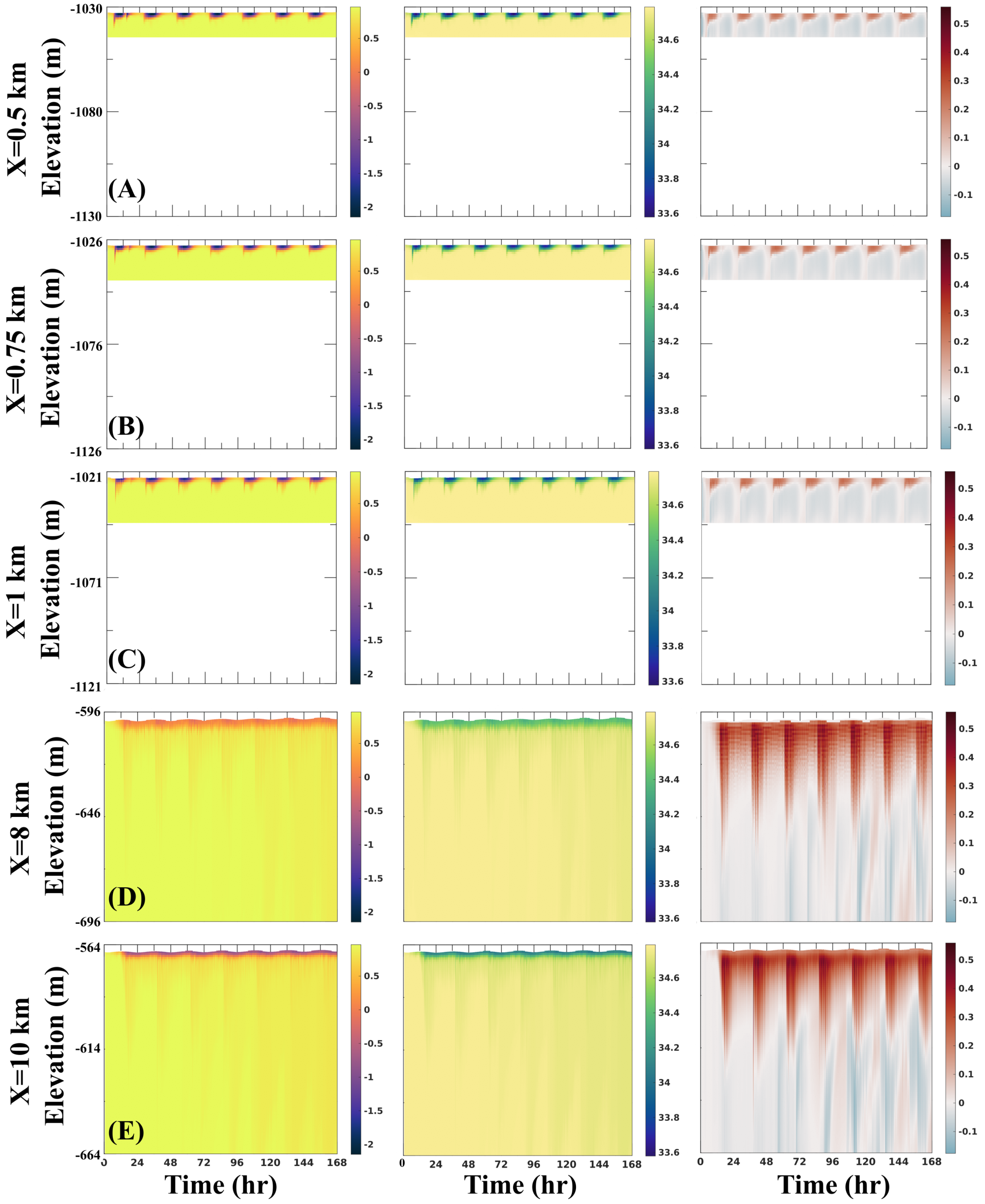

Figure. S5. Hovmöller diagrams of temperature (℃), salinity (psu), and speed (m/s) versus time (hours) in the ice shelf cavity of Thwaites Glacier Tongue (TGT). (A–E) diagrams at a distance, $X =$, 0.5, 0.75, 1, 8, and 10 km from the entrance of the IGZ, respectively, showing vertical profiles of temperature (left column), salinity (middle column) and speed (right column) within 100 m of the ice shelf base. **Positive speed is flow directed seaward, i.e., from the IGZ cavity toward the ice front**. White areas for X = 0.5, 0.75 and 1 km indicate no water. **The initial cavity height changes at x = 1 km, reflecting melting after the initial cycle. In contrast, later cycles exhibit small variations (0.2 m) close to the IGZ cavity entrance (x< 1 km), with visible tidal fluctuations in the cavity height near x = 10 km (D-E).**

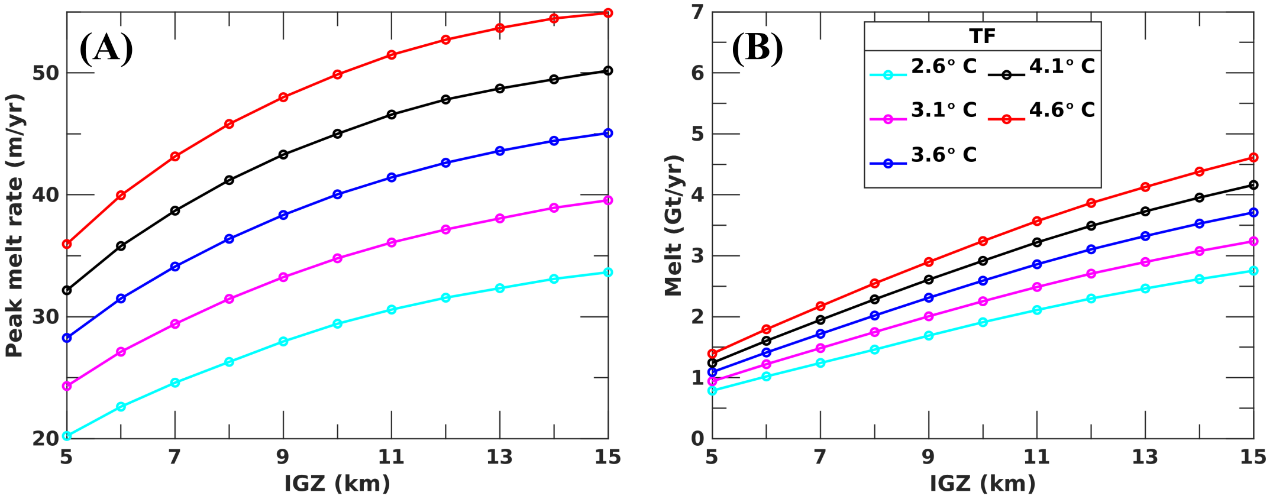

Figure. S6. Ice melt rate parameterization in the ice grounding zone (IGZ) of Thwaites Glacier Tongue (TGT). (A) peak melt rate, $\dot{m}_{max}$, at the cavity entrance and (B) integrated melt within the IGZ, M, as a function of IGZ length, L, and ocean thermal forcing, TF. Each symbol is one simulation with a linear fit between simulations. The model fit is: $\dot{m}_{max} = 4.865\ L^{0.4}\ TF^{0.916}$ and $M = 0.072\ L^{1.03}\ TF^{0.92}$.

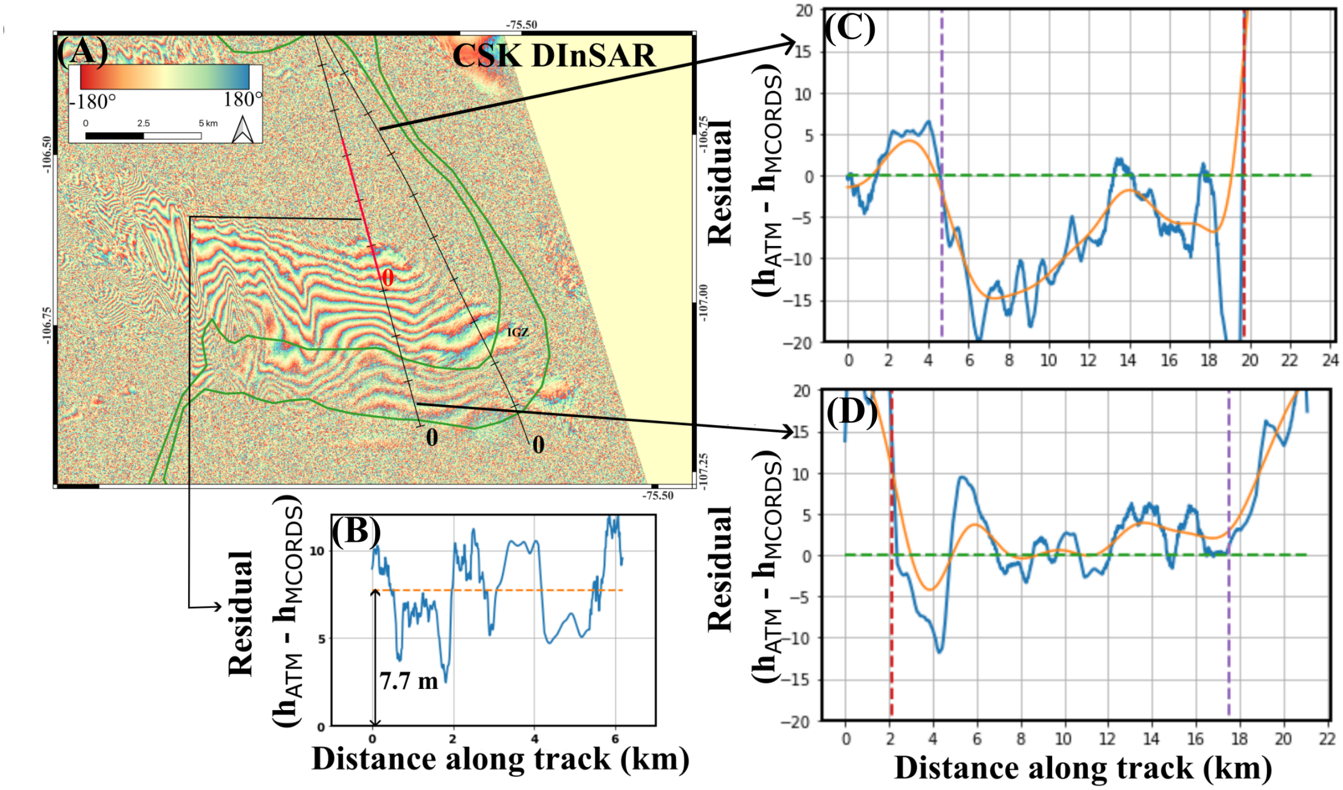

Figure. S7. Elevation residual from ATM laser altimetry minus elevation from ice-sounding MCoRDS radar data for Thwaites Glacier Tongue (TGT). (A) CSK differential interferogram combining data from December 2019 and May 2020 along with the Nov. 15, 2018 ATM/MCoRDS track (black solid), with tick marks every 2 km. Red line is used to calibrate ice thickness derived from ATM (i.e., HE). The 2018-2021 IGZ is green solid. Marking '0' is the origin of panels (B) and (D). (B) 2D cross-section of elevation residuals, with a mean of 7.7 m. (C, D) calibrated elevation residuals along the two segments shown in (A).

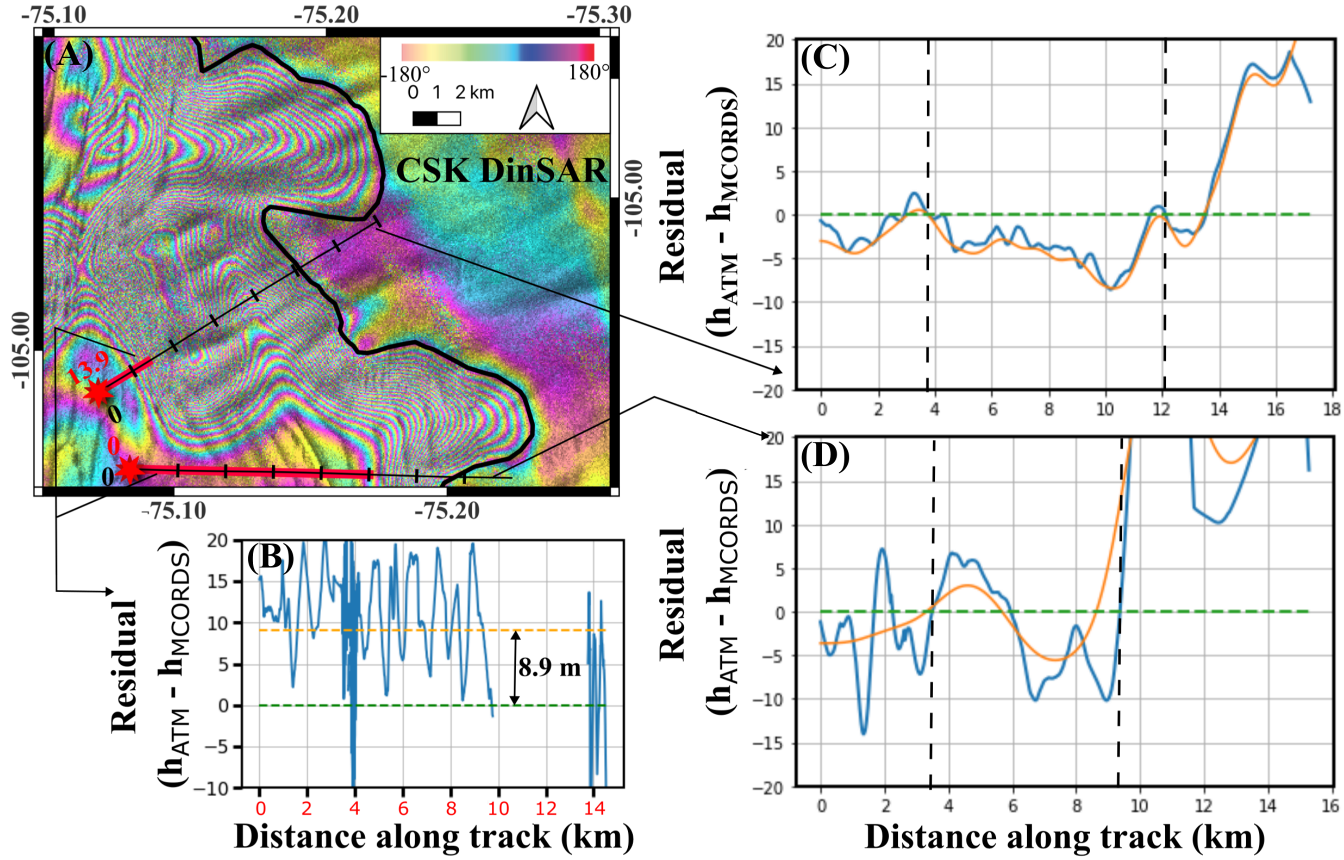

Figure. S8. Elevation residual from ATM laser altimetry minus elevation from ice-sounding MCoRDS radar data for Thwaites Eastern Ice Shelf (TEIS). (A) ERS-2 differential interferogram from April 4, 2011 along with Nov., 21 2013 ATM/MCoRDS track (black solid), with tick marks every 2 km. Red line is used to calibrate ice thickness derived from ATM. GL is a thick, black, solid line. Marking '0' in black is the origin of panels (C) and (D). Marking '0' in red is the origin of panel (B). (B) 2D cross-section of elevation residuals, with a mean of 8.9 m. (C, D) calibrated elevation residuals along the two segments shown in (A).

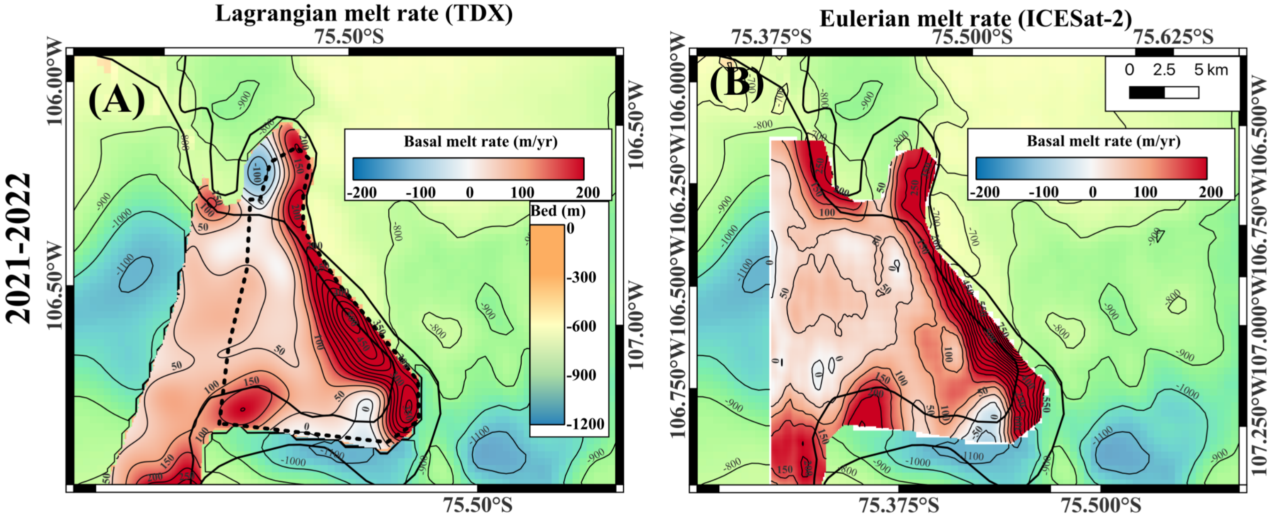

Figure. S9. Basal ice melt rates derived from remote sensing data on TGT in years 2021-2022. (A) Lagrangian-derived melt rates obtained combining TanDEM-X DEMs and MEaSUREs ice velocity. The average melt is 137 m/yr within the black dashed region. (B) Eulerian-derived melt rates obtained combining ICESat-2 ATL14/ATL15 elevation and MEaSUREs ice velocity. The average melt is 154 m/yr within the black dashed region. Background is BedMachine v3.7 bed topography with 100-m contour levels. ICEYE 2024 IGZ is a double black line in (A-B).

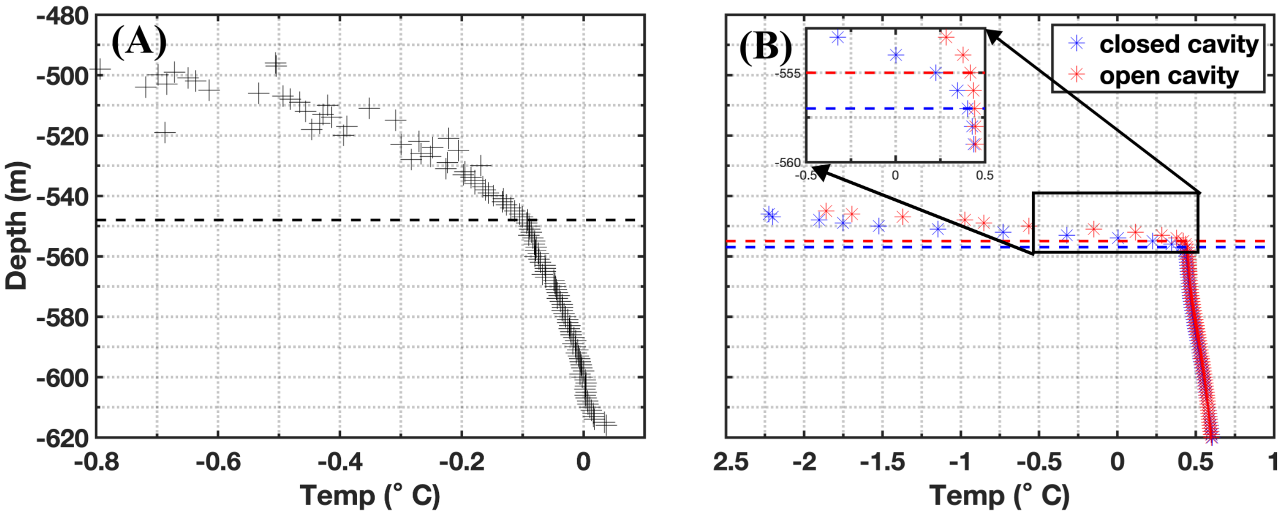

Figure. S10. Observed and modeled temperature profile beneath the ice shelf, averaged over the first 3 km near the IGZ cavity. (A) Temperature–depth profile from the IceFin AUV, vertically binned at 1 m resolution, with thermocline depth indicated as a black dotted line. (B) Temperature–depth profiles from MITgcm in the closed-cavity (blue) and open-cavity (red) configurations, with the corresponding thermocline depths (dotted lines). The inset in (B) is a zoomed-in view near the thermocline.

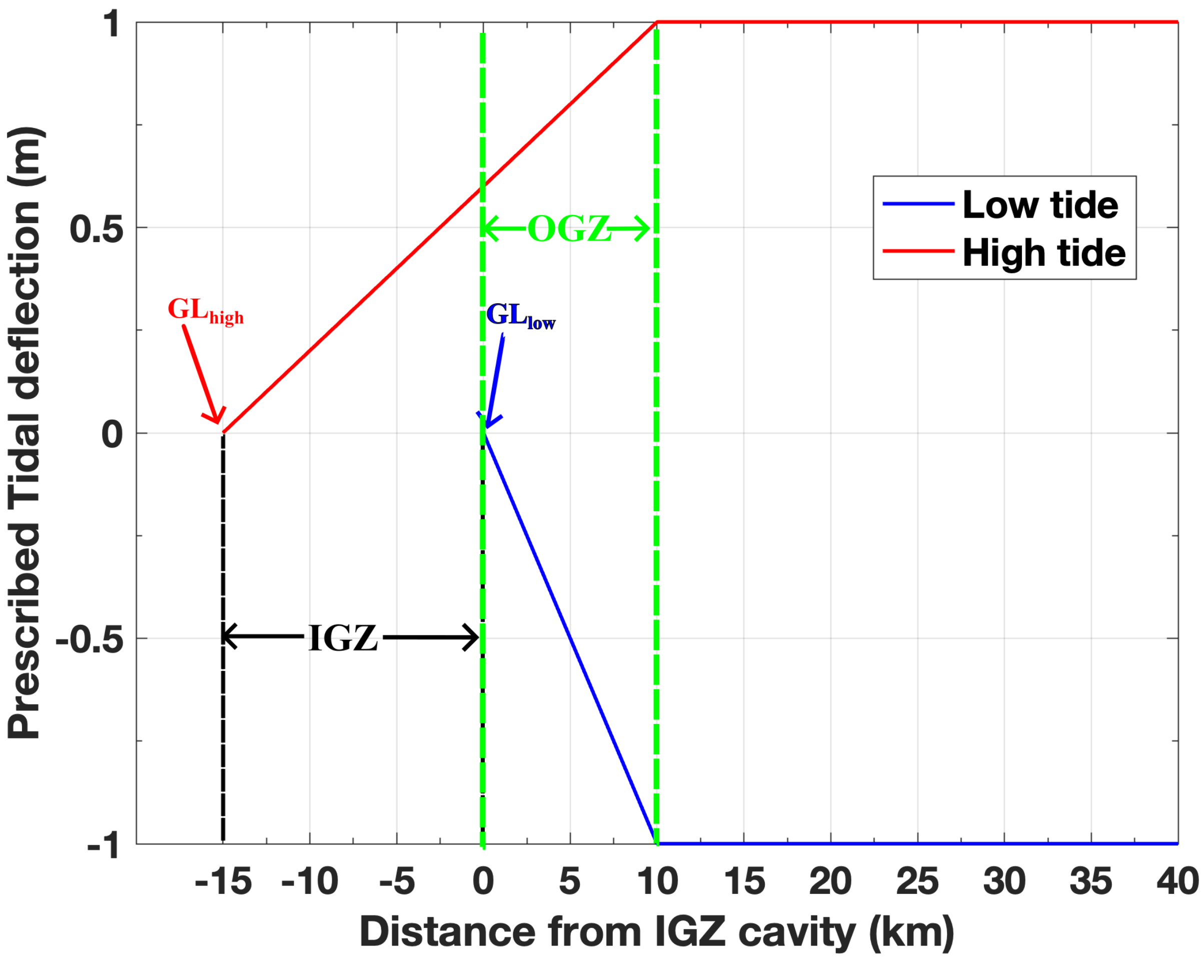

Figure. S11. Prescribed tidal deflection applied to MITgcm under low-tide (solid blue line) and high-tide configurations (solid green line). The locations of the IGZ (between the black dashed lines) and OGZ (between the green dashed lines) are labeled. In the low-tide configuration, a linear tidal deflection gradient is applied across the OGZ; in the high-tide configuration, the gradient spans both the OGZ and IGZ.

Table S1. Model parameters used in the MITgcm simulations.

Symbol	Description	Value	Unit
a	Freezing equation salinity coefficient	-5.73$\times10^{-2}$	℃/psu
b	Freezing equation constant coefficient	9.39$\times10^{-2}$	℃
c	Freezing equation pressure coefficient	-7.53$\times10^{-4}$	℃/Pa
c_d	Drag coefficient between ice and water	1.5$\times10^{-3}$	dimensionless
ρ_M	Seawater reference density	1027.5	kg/m^3
ρ_{ice}	Ice reference density	917.0	kg/m^3
C_{pI}	Specific heat capacity of ice	2009.0	J/(kg K)
C_{PM}	Specific heat capacity of mixed layer	3974.0	J/(kg K)
L_f	Latent heat fusion	3.34$\times10^{5}$	J/kg
S_{ice}	Ice salinity	0	psu
T_{ice}	Ice temperature	-20	℃
κ	Ice thermal conductivity	1.54$\times10^{-6}$	m^2/s
Γ_T	Turbulent heat transfer coefficient	0.01369	dimensionless
Γ_S	Turbulent salt transfer coefficient	4.42$\times10^{-4}$	dimensionless
γ_S	Salinity exchange coefficient	velocity dependent	m/s
γ_T	Temperature exchange coefficient	velocity dependent	m/s
u_*	Friction velocity	velocity dependent	m/s
U_M	Mixed layer velocity	model output	m/s
S	Seawater salinity	model output	psu
T	Seawater temperature	model output	℃
	Pressure-Salinity dependent		
T_f	Freezing temperature	model output dependent	℃
A_h	horizontal viscosity	0.3	m^2/s
A_r	vertical viscosity	2.8$\times10^{-4}$	m^2/s
κ_h	horizontal diffusivity of T,S	0	m^2/s
κ_r	vertical diffusivity of T,S	2.8$\times10^{-5}$	m^2/s
VISCA4	Horizontal Bi-harmonic viscosity	2.5	m^4/s
Δt	model time-step	0.5	s

Table S2. **Range of parameters tested in MITgcm simulations.**

Symbol	Minimum Value	Maximum Value
C_d	1$\times 10^{-3}$	2$\times 10^{-3}$
A_h	0.1	0.6
A_r	2.8$\times 10^{-3}$	2.8$\times 10^{-5}$
κ_r	2.8$\times 10^{-4}$	2.8$\times 10^{-6}$
